# End-to-End Image Demosaicking via Region-Level Non-Local Modeling and Residual Aggregation

**DOI:** 10.3390/s26092876

**Published:** 2026-05-05

**Authors:** Lingyun Wei, Han Liu

**Affiliations:** School of Automation and Information Engineering, Xi’an University of Technology, Xi’an 710048, China; weilingyun@stu.xaut.edu.cn

**Keywords:** image demosaicking, color filter array, non-local modeling, residual aggregation, image reconstruction

## Abstract

Image demosaicking aims to reconstruct a full-resolution color image from spatially sparse and interleaved color filter array observations. Despite the significant progress achieved by deep learning-based methods, existing approaches have not fully addressed the sampling-structure-constrained nature of demosaicking. In particular, four-channel half-resolution packing may disrupt the CFA spatial phase relationships, while local convolutions and global non-local matching struggle to model reconstruction-relevant cross-position dependencies. To address these issues, this paper proposes an end-to-end image demosaicking network with region-level non-local modeling and residual aggregation (RNRA-Net). Instead of packing Bayer RAW data into a four-channel half-resolution representation, RNRA-Net decomposes the original mosaic image into a three-channel representation at the original resolution, thereby preserving the spatial arrangement of CFA sampling. To capture structurally related information, a region-level non-local module is introduced to compute feature correlations within spatially bounded regions, enabling aggregation of reconstruction-relevant contextual information. In addition, a residual aggregation module is developed to explicitly collect and refine early residual compensation features, facilitating the recovery of edges, textures, and high-frequency details. Extensive experiments on benchmark and high-resolution datasets demonstrate the effectiveness of RNRA-Net.

## 1. Introduction

Image demosaicking is a crucial initial step in the image signal processing (ISP) pipeline of digital cameras. Its purpose is to rebuild a full-resolution color image from sparse single-channel observations captured by a color filter array (CFA). The Bayer CFA is the most widely used CFA pattern [1]. Since only one color component is recorded at each pixel location, a large amount of scene information is lost during sampling, making demosaicking a highly ill-posed image reconstruction problem [2]. This difficulty is particularly evident in edge regions, textured areas, and high-frequency details, where incomplete sampling often causes false colors, zipper artifacts, structural blurring, and detail loss. These errors may further propagate through subsequent ISP stages and degrade the final image quality. Therefore, recovering structurally faithful and chromatically consistent color images from highly sparse and incomplete observations remains a fundamental problem in image demosaicking.

Existing demosaicking methods can be broadly classified into either conventional interpolation-based or deep learning-based approaches. Conventional methods usually rely on local directional interpolation, gradient priors, or inter-channel correlations, such as bilinear interpolation [3], constant-hue-based interpolation [4], and residual interpolation methods [5,6,7]. Although these methods can preserve edge structures to some extent, they rely on fixed rules or handcrafted assumptions and thus have limited capacity to handle complex textures and nonlinear color relationships.

In recent years, deep learning-based methods have significantly advanced demosaicking performance and gradually become the dominant paradigm. Benefiting from the rapid development of deep learning over the past decade and its success across a wide range of tasks [8,9,10,11,12,13,14], existing deep demosaicking methods can be roughly grouped into three categories. The first category is multi-stage demosaicking methods [15,16,17,18]. Hou et al. proposed a two-stage demosaicking model (DTDeMo) [18], which decomposes demosaicking into interpolation and enhancement stages, implemented by a convolutional interpolation block and a residual enhancement module, respectively. While this design explicitly decomposes the reconstruction process, its performance largely depends on the quality of the preceding interpolation stage. Errors introduced in the early stage may propagate to the subsequent enhancement stage and limit the final reconstruction quality. The second category is end-to-end demosaicking methods [19,20,21,22,23,24,25]. Among them, Wang et al. [25] adopted a U-Net++-style encoder–decoder architecture for end-to-end reconstruction, where the Bayer mosaic is rearranged into a four-channel RGGB representation to improve modeling flexibility. A knowledge learning-based demosaicking model for adaptive patterns (KLAP) [24], on the other hand, handles both Bayer and non-Bayer patterns in a unified framework via CFA-adaptive filtering and meta-learning, while also accounting for sensor artifacts in real RAW images. Although these methods bypass explicit stage decomposition and learn the input–output mapping directly, the packed input representation weakens the explicit spatial constraints encoded in the original CFA pattern. The third category is joint denoising and demosaicking methods [26,27]. For instance, Zhang et al. [26] proposed the deep plug-and-play image restoration (DPIR) framework, which integrates residual blocks into a U-Net for multiple image restoration tasks, and MGCC-JDD [27] performs joint denoising and demosaicking via spatially adaptive cross-channel fusion. These methods jointly model noise suppression and color reconstruction, or leverage deep denoising priors to solve demosaicking under complex degradations. However, their feature extraction still mainly relies on local convolutions or generic restoration priors. In demosaicking, valid pixels from different color channels are spatially sparse and discontinuous. Purely local operations are therefore insufficient to fully exploit preserved observations or capture long-range dependencies, which limits the recovery of complex edges and fine textures. Overall, despite steady progress in network architectures and reconstruction paradigms, existing methods still fall short in capturing cross-position correlations and preserving the structural constraints inherent in the original CFA.

Despite the significant progress achieved by deep learning-based demosaicking methods, the key modeling requirements induced by CFA sampling have not been fully addressed. Unlike generic image restoration, demosaicking is essentially a sampling-structure-constrained reconstruction problem, where valid color observations are spatially sparse, interleaved, and phase-dependent. Therefore, an effective model should not only learn a strong mosaic-to-RGB mapping, but also preserve the original CFA geometry. Simply packing Bayer measurements into a four-channel half-resolution representation may facilitate network processing, but it can weaken the pixel-level spatial phase relationships inherent in the CFA pattern. Moreover, the recovery of missing color components depends not only on adjacent pixels but also on structurally related positions with consistent edge orientations, texture patterns, or CFA phase relationships. Purely local convolutions are limited in explicitly capturing such cross-position dependencies, whereas unrestricted global non-local matching may introduce misleading chromatic responses from structurally irrelevant regions, leading to color contamination near edges and repetitive textures.

Motivated by the above observations, we propose an end-to-end image demosaicking network with region-level non-local modeling and residual aggregation (RNRA-Net). Instead of packing Bayer RAW data into a four-channel half-resolution representation, RNRA-Net decomposes the mosaic image into a three-channel representation at the original resolution, thereby maintaining the spatial arrangement of the CFA sampling pattern. To model cross-position dependencies in a reconstruction-relevant manner, we introduce a region-level non-local module that computes feature correlations within spatially bounded regions. This design enables adaptive aggregation of structurally related information while suppressing unreliable global matching. Furthermore, we develop a residual aggregation module to explicitly collect and refine early residual compensation cues, facilitating the recovery of edges, textures, and high-frequency details. To further clarify the mechanism-level distinction between RNRA-Net and existing approaches, Table 1 compares representative demosaicking methods with respect to input representation, correlation modeling, and high-frequency feature usage. RNRA-Net differs from existing methods by jointly preserving CFA sampling geometry, constraining non-local correlation to reconstruction-relevant regions, and explicitly aggregating residual compensation cues.

The main contributions of this paper are as follows:

- In this paper, we propose an end-to-end image demosaicking network with region-level non-local modeling and residual aggregation (RNRA-Net) that jointly considers sampling-geometry preservation, constrained structural correlation, and residual compensation modeling.
- A region-level non-local module is developed to bridge the gap between local convolution and unrestricted global matching, enabling reconstruction-relevant feature interaction while reducing irrelevant chromatic interference.
- We propose a residual aggregation strategy that fully exploits the relatively pure residual cues in early-stage features, thereby improving feature extraction efficiency and enhancing high-frequency detail recovery.
- Extensive experiments demonstrate the effectiveness of RNRA-Net on both benchmark and high-resolution datasets.

The proposed method is described in detail in Section 2, Section 3 shows a series of experiments to verify the performance of the model, and finally, this paper discusses and summarizes the proposed method in Section 4 and Section 5.

## 2. Methodology

### 2.1. Network Architecture

**Figure 1:**
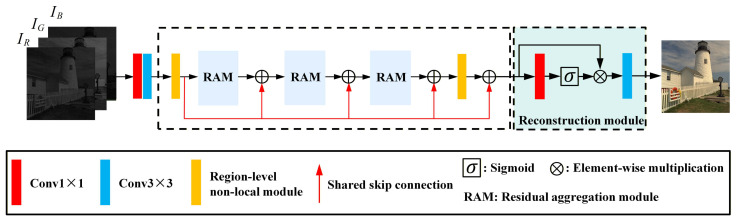
The general network structure of RNRA-Net.

The overall architecture of RNRA-Net is shown in Figure 1. The proposed network consists of three parts: a shallow feature extraction module, a backbone module, and a reconstruction module. Given an input Bayer mosaic image, RNRA-Net first converts it into a three-channel representation at the original resolution. This preserves the spatial arrangement of the CFA pattern and serves as the input to the subsequent end-to-end reconstruction process.

The shallow feature extraction module uses a 1×1 convolution followed by a 3×3 convolution to extract the initial low-level representations from the input mosaic image. The backbone module places two region-level non-local modules at the beginning and the end, with three residual aggregation modules cascaded in between. This design allows the network to capture regional contextual dependencies and reuse early residual compensation cues. In addition, a shared skip connection is introduced in the backbone to bypass low-frequency components, allowing the network to focus on reconstructing high-frequency structures and fine texture details. Finally, the reconstruction module further refines the learned features and generates the output RGB image.

Let $I_{m}$ denote the input mosaic image. The shallow features $F_{s}$ are first extracted as

(1) $$F_{s}=H_{s}\left(I_{m}\right)=\phi_{3\times 3}\;\left(\phi_{1\times 1}\left(I_{m}\right)\right),$$

where $\phi_{1\times 1}\left(\cdot \right)$ and $\phi_{3\times 3}\left(\cdot \right)$ denote the 1×1 and 3×3 convolution operations, respectively.

The extracted shallow features are then fed into the backbone for deep feature $F_{d}$ learning:

(2) $$F_{d}=H_{b}\left(F_{s}\right),$$

where $H_{b}\left(\cdot \right)$ denotes the nonlinear mapping learned by the backbone. Specifically, the region-level non-local modules model feature correlations within appropriate neighborhoods to enlarge the effective receptive field and capture informative contextual cues, while the residual aggregation modules repeatedly preserve and exploit relatively pure residual information to improve feature extraction efficiency. The shared skip connection further guides the network to bypass low-frequency components and focus on reconstructing detailed textures.

The deep features are subsequently sent to the reconstruction module for final refinement. To emphasize informative structures, a simplified channel-spatial attention layer is introduced. As shown in Figure 1, an attention map is first generated by a 1×1 convolution followed by a sigmoid activation, and the refined RGB image $\hat{I}$ is then estimated by a 3×3 convolution:

(3) $$A=\sigma \;\left(\phi_{1\times 1}\left(F_{d}\right)\right),\;\hat{I}=R\left(F_{b}⊙A\right),$$

where σ(·) denotes the sigmoid function, ⊙ denotes element-wise multiplication, and R(·) denotes the reconstruction operation implemented by a 3×3 convolution.

During training, the network parameters are optimized by minimizing the $\ell_{2}$ loss:

(4) $$L\left(\Theta \right)={∥F\left(I_{m};\Theta \right)-I_{gt}∥}_{2}^{2},$$

where Θ denotes the learnable parameters, $I_{gt}$ is the ground-truth image corresponding to $I_{m}$, and F(·) represents RNRA-Net.

### 2.2. Region-Level Non-Local Module

In image demosaicking, the recovery of missing color components depends not only on local interpolation cues but also on cross-position structural relationships, such as texture repetitiveness, edge continuity, and similarity among spatially separated patterns. Because CFA sampling produces spatially sparse and interleaved observations, the missing value at a target position is often related to other positions that share similar local structures. Conventional convolutions aggregate features progressively within a limited receptive field and are therefore effective for local pattern extraction. However, they cannot explicitly model direct interactions between distant yet structurally correlated positions, which limits their ability to recover fine edges, repeated textures, and other high-frequency details. Non-local operations provide a natural solution by computing responses based on feature similarity across spatial positions, thereby capturing long-range dependencies beyond local convolution. However, directly applying global non-local computation to high-resolution feature maps is suboptimal for demosaicking. In addition to the high computational and memory cost, global matching may introduce responses from regions that are weakly related to the current reconstruction area, which can dilute useful structural cues and disturb local detail recovery. This issue is especially critical for demosaicking, where accurate reconstruction is highly sensitive to edge orientation, local continuity, and the spatial relationships among similar CFA sampling patterns. Region-constrained non-local interaction offers a better balance between local convolution and unrestricted global matching. Motivated by this observation, we design a region-level non-local module that performs non-local modeling within partitioned local regions, thereby reducing computational complexity and extracting more relevant contextual information for demosaicking.

**Figure 2:**
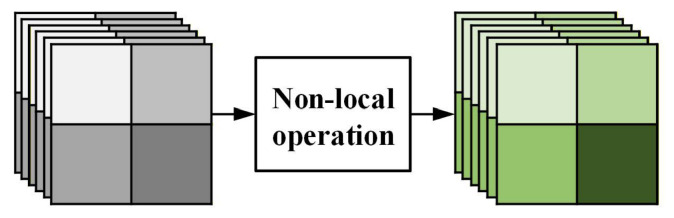
Details of the region-level non-local module.

As shown in Figure 2, the input feature map is first divided along the spatial dimensions into four non-overlapping sub-regions in a 2×2 manner. A non-local operation is then performed independently within each sub-region. In other words, each position only interacts with other positions inside the same region rather than with all positions in the entire feature map. After region-wise non-local enhancement, all sub-regions are reassembled according to their original spatial layout to recover the complete feature representation.

**Figure 3:**
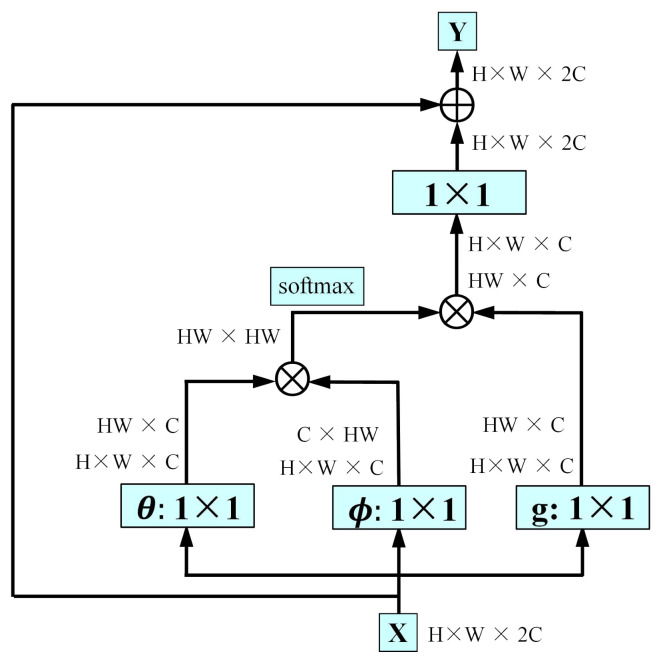
Details of the non-local operation module.

The form of non-local operations within the region is shown in Figure 3. For a given position *i* within a local region $\Omega_{r}$, the non-local response is computed as

(5) $$y_{i}=\frac{1}{C(x)}\sum_{j\in \Omega_{r}}f\left(x_{i},x_{j}\right)\;g\left(x_{j}\right),$$

where $x_{i}$ and $x_{j}$ denote the input features at positions *i* and *j*, respectively. C(x) is a normalization factor. g(·) is a linear embedding function, and f(·,·) measures the pairwise correlation between positions *i* and *j*. In this work, f(·,·) is implemented by an embedded Gaussian function:

(6) $$f\left(x_{i},x_{j}\right)=\exp\;\left(\theta {\left(x_{i}\right)}^{⊤}\phi \left(x_{j}\right)\right),$$

where θ(·) and ϕ(·) are learnable linear transformations. The output $y_{i}$ denotes the aggregated feature at position *i*. Compared with standard convolution, which relies on fixed local kernels, the proposed region-level non-local module enables each position to adaptively gather information from all positions within its region. As a result, it enhances contextual modeling while avoiding irrelevant interference and additional overhead caused by global non-local computation.

### 2.3. Residual Aggregation Module

**Figure 4:**
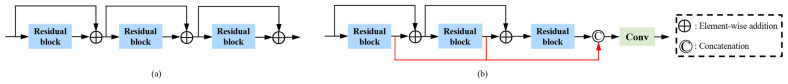
The feature extraction method of traditional residual and residual aggregation ((**a**) traditional residual, (**b**) residual aggregation).

**Figure 5:**
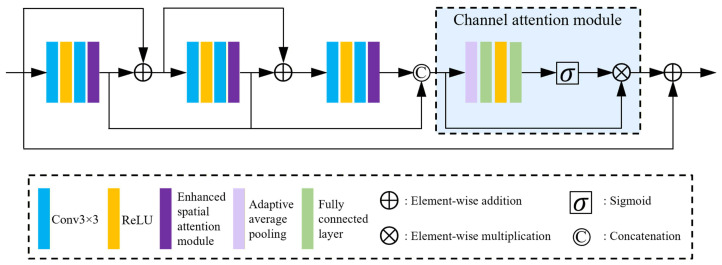
The structure of the residual aggregate module.

Residual connections are effective for preserving detailed information, and residual branches at different depths may capture complementary spatial structures. As the network becomes deeper, features undergo more convolutional transformations, making early residual cues progressively less distinguishable. As a result, the learning of residual features in each branch can be easily weakened or overlooked. To address this issue, RNRA-Net introduces a residual aggregation strategy and develops a residual aggregation module to improve feature extraction efficiency. As illustrated in Figure 4a, conventional residual feature extraction usually improves performance by stacking more residual blocks. In such a design, the features generated by early blocks must travel through a long propagation path before reaching later layers. After repeated element-wise additions and convolutions, these features are quickly merged with identity features and transformed into more complex representations. As a result, highly informative early residual cues are only locally exploited, and later layers mainly receive heavily fused features rather than relatively clean residual information from earlier layers, which may hinder final reconstruction quality. To overcome this limitation, RNRA-Net adopts the residual aggregation strategy shown in Figure 4b. Specifically, three residual blocks are stacked. Different from conventional residual learning, the third residual block does not simply follow the previous residual propagation path. Instead, the outputs of the preceding residual branches are directly aggregated at the output position of the third block. A subsequent convolution is then applied to integrate and refine these aggregated features, yielding more representative feature responses. As shown in Figure 5, the residual aggregation module first exploits relatively pure residual features through aggregation, then employs an enhanced spatial attention (ESA) module to emphasize informative spatial distributions, and finally introduces a channel attention module as a complementary mechanism to further highlight important channel-wise responses.

**Figure 6:**
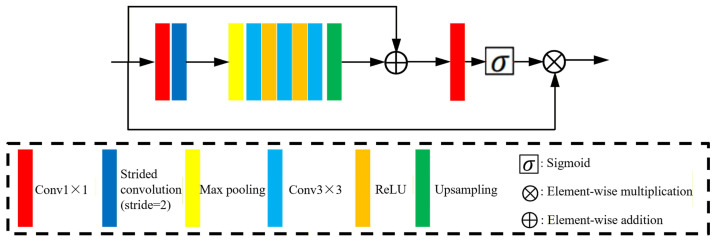
The structure of the enhanced spatial attention module.

Residual features extracted by different residual blocks can characterize different aspects of spatial content, but these residual cues are often insufficiently emphasized. To further improve the representational capability of the module, we incorporate a spatial attention mechanism to enhance the spatial distribution of residual features. To reduce the cost of spatial attention, an ESA module is adopted, as shown in Figure 6. The ESA module enlarges the receptive field through strided convolution and max pooling, while reducing the channel dimensionality in the front part of the module to focus the features on more informative spatial content.

Given an input feature $F_{\mathrm{in}}$, the residual features extracted through a sequence of convolutions and ESA operations are formulated as

(7) $$\left\{\begin{array}{cc} F_{1} & =H_{s1}\left(Conv_{1}\left(F_{\mathrm{in}}\right)\right),\\ F_{2} & =H_{s2}\left(Conv_{2}\left(F_{1}⊕F_{\mathrm{in}}\right)\right),\\ F_{3} & =H_{s3}\left(Conv_{3}\left(F_{2}⊕F_{1}⊕F_{\mathrm{in}}\right)\right),\\ F_{res} & =Concat(F_{1},F_{2},F_{3}), \end{array}\right.$$

where $F_{1}$, $F_{2}$, and $F_{3}$ are the residual features extracted from the first, second, and third branches, respectively, and $F_{res}$ is the aggregated residual representation. $H_{si}\left(\cdot \right)$ denotes the *i*-th enhanced spatial attention (ESA) operation, $Conv_{i}\left(\cdot \right)$ denotes the *i*-th convolution–ReLU–convolution transformation, ⊕ denotes element-wise addition, and Concat(·) denotes channel-wise concatenation.

To further complement spatial attention, a channel attention mechanism is introduced to emphasize informative channel responses, as shown in Figure 5. Specifically, adaptive average pooling is first used to compress the spatial information into a channel descriptor, and then two fully connected layers, followed by a sigmoid activation, are employed to obtain the channel weights:

(8) $$z=H_{pooling}\left(F_{\mathrm{res}}\right),$$

(9) $$w=Sigmoid\left({FC}_{2}\;\left(ReLU\left({FC}_{1}\left(z\right)\right)\right)\right),$$

where $H_{pooling}$ denotes adaptive average pooling, ${FC}_{1}\left(\cdot \right)$ and ${FC}_{2}\left(\cdot \right)$ denote fully connected layers. The refined feature is obtained by assigning the channel weights to the corresponding channels of the aggregated residual feature:

(10) $$\tilde{F}=w⊙F_{res},$$

where ⊙ denotes element-wise multiplication. Finally, a long skip connection is used to add the refined feature to the input feature, producing the output of the residual aggregation module:

(11) $$F_{\mathrm{out}}=F_{\mathrm{in}}+\tilde{F}.$$

In summary, the proposed module enhances residual compensation by aggregating early-stage residual features and refining them with spatial and channel attention. The combination of spatial and channel attention further guides the network to focus on severely degraded regions from both spatial and channel perspectives, thereby facilitating accurate reconstruction of diverse texture details.

## 3. Experiment

This section conducts multiple experiments to validate the RNRA-Net’s performance. Section 3.1 details the datasets utilized and provides experimental specifics. Section 3.2 and Section 3.3 demonstrate the reconstruction performance of RNRA-Net on benchmark and high-resolution datasets. Section 3.4 outlines a series of ablation experiments. Section 3.5 provides the complexity analysis.

### 3.1. Implementation Setting

**Dataset**. We use 800 training images and 100 validation images from DIV2K [28], together with 2650 high-resolution images from Flickr2K [29], to train RNRA-Net. For evaluation, two widely used benchmark datasets, Kodak [30] and McMaster [31] were used. The Kodak dataset contains 24 outdoor images originally captured on film, while the McMaster dataset consists of 18 high-resolution images with richer color and texture variations. Although Kodak has been widely adopted in previous demosaicking studies and remains useful for both comparison with existing methods and ablation analysis, its relatively limited spatial resolution and small number of images may not fully reflect the challenges of modern high-resolution camera images. Therefore, we further include City100 [32] as a complementary benchmark. Originally introduced for image super-resolution, City100 contains 100 real photographs captured by two different devices, namely a Nikon D5500 DSLR camera and an iPhone X smartphone, and thus provides a complementary high-resolution benchmark for evaluating demosaicking performance on images with complex scene structures and dense textures.

**Data preprocessing**. For all datasets used in this work, including Kodak, McMaster, and City100, the demosaicking input–target pairs are generated from the available full-color RGB images using a unified preprocessing pipeline. Each RGB image is first converted to the standard RGB color space, normalized to the range of [0,1], and cropped by one pixel along the height or width dimension when necessary to match the Bayer sampling grid. The processed RGB image is used as the ground-truth image. The mosaic input is then generated by applying the standard RGGB Bayer color filter array pattern, where only one color component is retained at each spatial location. The same Bayer sampling protocol is used for both training and testing. For RNRA-Net, the resulting Bayer observation is converted into a three-channel original-resolution representation that preserves the spatial arrangement of CFA samples. For the compared methods, the same Bayer observation is used and converted to the corresponding input format required by each method. In this way, all methods are evaluated under the same Bayer pattern, preprocessing pipeline, and ground-truth definition. Since Kodak, McMaster, and City100 provide RGB images rather than paired sensor RAW observations, the experiments are conducted under a controlled synthetic Bayer sampling setting. Therefore, the reported results evaluate demosaicking performance from sparse RGGB CFA observations, but do not explicitly model sensor-specific RAW noise or camera ISP operations such as black-level correction, white balance, and color correction.

**Implementation**. During training, a total of 534,287 non-overlapping image patches of size 128×128 were extracted from the DIV2K [28] and Flickr2K [29] datasets to construct the training set. The proposed RNRA-Net was implemented in PyTorch 1.7.1 [33] and optimized using Adam [34], with $\beta_{1}=0.9$ and $\beta_{2}=0.999$. The initial learning rate was set to $1\times 10^{-4}$ and decayed by a factor of 0.5 every 10 epochs. The model was trained for 30 epochs with a batch size of 8 on an NVIDIA GeForce RTX 2080 GPU (Nvidia, Santa Clara, CA, USA) with 8 GB memory. The model achieving the best validation performance was selected for testing. To verify the superiority of the proposed RNRA-Net, we compared it with five existing demosaicking methods, including one traditional method, i.e., bilinear [3], and four deep learning-based methods, namely U-Net++ [25], DPIR [26], KLAP [24], and DTDeMo [18]. In addition to the two widely used objective evaluation metrics, namely peak signal-to-noise ratio (PSNR) and structural similarity index (SSIM), we further incorporate learned perceptual image patch similarity (LPIPS) [35] and the CIEDE2000 color-difference formula [36] proposed by the International Commission on Illumination, to provide a more comprehensive assessment of perceptual quality and chromatic fidelity. To reduce the influence of random initialization, all reported experimental results are averaged over five independent runs with different random seeds. To ensure a fair comparison, all methods were evaluated under the same data construction and evaluation protocol, including the same RGB reference images, synthetic RGGB Bayer sampling process, preprocessing pipeline, ground-truth definition, and evaluation metrics. The generated Bayer observations were then converted into the input format required by each method’s original design or released implementation. As summarized in Table 2, different methods may adopt distinct input representations, architectures, and method-specific hyperparameters; therefore, we did not force all internal settings to be identical. Instead, the common data construction and evaluation protocol was kept consistent, while method-specific settings were configured according to the corresponding original papers or released implementations.

### 3.2. Evaluation on Benchmark Datasets

Table 3 shows the quantitative comparisons on the Kodak and McMaster datasets (the best and suboptimal results are highlighted in bold and underlined, respectively). Overall, RNRA-Net achieves the best comprehensive performance. On Kodak, RNRA-Net attains the highest PSNR and SSIM, along with the lowest CIEDE2000, while its LPIPS is nearly identical to the best result. On the more challenging McMaster dataset, our method ranks first across all four metrics. Compared with U-Net++, DPIR, and KLAP, these methods mainly emphasize local convolutional feature reuse, generic restoration-prior modeling, and unified CFA representation, respectively, but they do not simultaneously account for the constrained-region long-range dependency modeling, high-frequency residual preservation, and color transition recovery required by demosaicking. Although DTDeMo combines interpolation-guided reconstruction with Transformer-based enhancement and achieves the best LPIPS on Kodak, its overall pipeline still follows a two-stage local compensation strategy. In contrast, RNRA-Net, through the collaborative design of the region-level non-local modules and residual aggregate modules, integrates non-local contextual information with high-frequency residual features more effectively. As a result, compared with DTDeMo on Kodak, RNRA-Net further improves PSNR by 1.17 dB and SSIM by 0.0020, while reducing CIEDE2000 by 0.2976. On McMaster, it also surpasses the second-best results by 0.11 dB in PSNR, 0.0110 in SSIM, 0.0003 in LPIPS, and 0.1098 in CIEDE2000. These results demonstrate that the proposed architecture achieves a more balanced optimization among pixel-wise fidelity, structural preservation, and color restoration.

**Figure 7:**
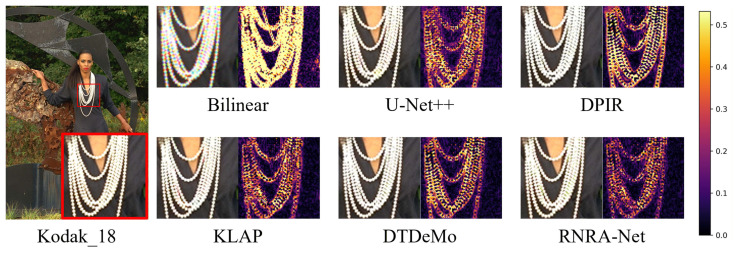
The reconstruction result and mean absolute RGB error maps of different demosaic methods on Kodak_18. Use a red box to highlight the local magnified view.

**Figure 8:**
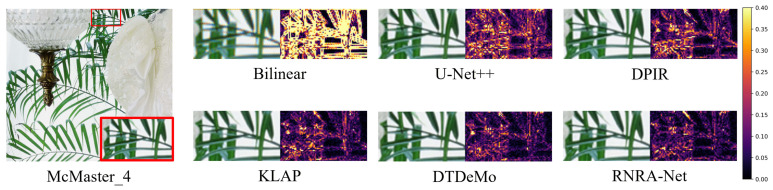
The reconstruction result and mean absolute RGB error maps of different demosaic methods on McMaster_4. Use a red box to highlight the local magnified view.

To further validate the superiority of the RNRA-Net on benchmarks, we provide visual comparisons and corresponding color-difference maps on Kodak and McMaster in Figure 7. In the Kodak_18 example, RNRA-Net reconstructs the fine necklace structures with clearer boundaries, higher geometric continuity, and more faithful local contrast. By comparison, bilinear suffers from severe blurring and zipper artifacts, while U-Net++, DPIR, and KLAP still exhibit noticeable structural distortion and residual color deviation around the high-frequency regions. Although DTDeMo produces relatively sharp results, slight reconstruction inconsistencies can still be observed along the curved necklace edges. In contrast, RNRA-Net preserves these delicate structures more accurately and yields a result that is visually closer to the ground truth. Similar trends can also be observed in the McMaster_4 example, as shown in Figure 8. For the thin plant edges and repeated stripe-like patterns, the proposed method produces cleaner boundaries and more natural color transitions, whereas the competing methods either introduce false colors or fail to fully recover the subtle high-frequency details. These observations are further confirmed by the color-difference maps. Specifically, our method exhibits weaker error responses and fewer concentrated high-error regions, indicating that it can more effectively suppress local reconstruction errors and chromatic distortions. Such consistent advantages in both visual appearance and error distribution demonstrate the superior capability of the proposed architecture in preserving fine structures and maintaining color fidelity on demosaicking benchmarks.

### 3.3. Evaluation on High-Resolution Dataset

Table 4 reports the quantitative comparison of the City100 dataset. Overall, the proposed RNRA-Net achieves consistently strong performance, demonstrating its effectiveness in handling high-resolution scenes with dense textures and structural details. Compared with bilinear interpolation, learning-based methods show better performance, indicating the limitation of fixed local interpolation in handling complex edges and color variations. U-Net++ improves efficiency with a lightweight convolutional design, while DPIR benefits from an iterative deep denoiser prior. KLAP and DTDeMo further improve reconstruction quality through CFA-aware modeling and two-stage interpolation-enhancement, respectively. In contrast, RNRA-Net still achieves the best performance, showing that region-level non-local modeling and residual aggregation can better exploit structural correlations and residual cues. This mainly benefits from its region-level non-local module, which captures reliable structural interactions, and its residual aggregation strategy, which further enhances high-frequency detail recovery.

**Figure 9:**
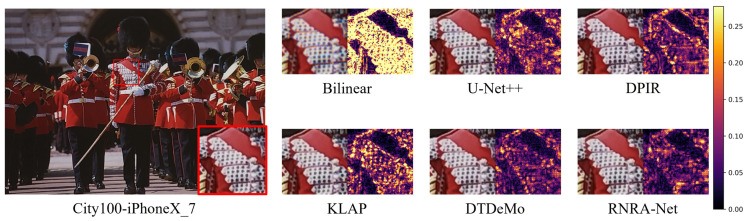
The reconstruction result and mean absolute RGB error maps of different demosaic methods on City100-iPhoneX_7. Use a red box to highlight the local magnified view.

Figure 9 provides a visual comparison of the City100 dataset, offering further insight into the behavior of different demosaicking methods on high-resolution images. In the City100-iPhoneX_7 example, bilinear interpolation produces severe blurring and aliasing artifacts, causing the fine lattice pattern in the uniform region to be largely indistinguishable. U-Net++ partially suppresses the blurring effect, but the recovered grid structure remains incomplete, and noticeable chromatic deviations can still be observed around the textured area. DPIR yields slightly sharper local details, but the repetitive pattern is not reconstructed in an orderly manner, and residual color distortions persist in the high-frequency region. KLAP better preserves the global structural layout and produces a more recognizable lattice pattern, although subtle irregularities remain in the periodic texture. DTDeMo further enhances local sharpness, particularly around the white textured region, but the reconstructed pattern still lacks sufficient spatial consistency across adjacent areas, with slight false-color artifacts appearing near the red–white transition. By contrast, RNRA-Net reconstructs the lattice pattern with improved regularity and continuity, while maintaining a cleaner and more faithful boundary between the red cloth region and the white textured structure.

### 3.4. Ablation Experiments

Table 5 presents the ablation results of different components on the Kodak dataset. The baseline model only relies on basic convolutional feature extraction and reconstruction, and thus has limited ability to preserve residual compensation cues and model structural dependencies. Introducing the residual aggregation module improves the utilization of intermediate residual features, which is beneficial for recovering edges, textures, and color transitions. This is because early residual cues in demosaicking often contain useful high-frequency compensation information, but they may be weakened when propagated through ordinary stacked layers. The region-level non-local module provides an additional ability to capture structurally related information within constrained spatial regions. This is helpful for repetitive textures and continuous edge structures, where local convolution alone may be insufficient to infer missing color components. Nevertheless, the ablation results also indicate that non-local modeling should be combined with residual aggregation and skip connection to achieve its full effect. Without sufficient residual refinement and stable information propagation, contextual interaction alone cannot fully exploit high-frequency reconstruction cues. The shared skip connection plays a complementary role by allowing low-frequency information to bypass the backbone, so that the main nonlinear path can focus more on residual and high-frequency reconstruction. When all components are integrated, RNRA-Net achieves the best overall reconstruction fidelity and structural consistency, demonstrating that residual aggregation, shared skip connection, and region-level non-local modeling contribute from different perspectives and jointly improve demosaicking performance. It is worth noting that the LPIPS value of the complete RNRA-Net is marginally higher than that of Model 4, but the difference is very small. This can be interpreted as a minor perceptual fluctuation rather than an obvious degradation in reconstruction quality. Since LPIPS relies on deep feature responses, it may be sensitive to subtle local texture variations. The region-level non-local module improves spatial consistency and pixel-level fidelity by enhancing structurally related feature interactions, but it may also slightly alter local perceptual responses. Therefore, the small LPIPS variation is acceptable, and the complete RNRA-Net still achieves the most balanced performance in terms of fidelity, structural preservation, and perceptual quality.

Table 6 further compares the reconstruction performance obtained with different numbers of residual aggregate blocks on the Kodak dataset. As the number of blocks increases from one to three, the performance improves consistently, with PSNR and SSIM gradually increasing and LPIPS correspondingly decreasing. This trend indicates that stacking multiple aggregate residual blocks is beneficial for progressively enhancing feature representation and high-frequency detail recovery. In particular, the best performance is achieved when three blocks are adopted, suggesting that this configuration provides a favorable balance between representation capacity and reconstruction efficiency. When the number of blocks is further increased to four, the performance slightly degrades on all three metrics. This observation implies that simply deepening the aggregation stage does not necessarily lead to further gains. Instead, excessive stacking may introduce redundant feature transformation and weaken the effectiveness of residual aggregation. Therefore, using three aggregate residual blocks is the most appropriate setting for the proposed network.

To further verify the benefit of preserving the original CFA spatial structure, we conduct an ablation study by replacing the proposed original-resolution CFA representation with the widely used packed RGGB representation, while keeping the rest of the network architecture and training settings unchanged. As shown in Table 7, the proposed representation consistently outperforms the packed RGGB input across all evaluation metrics. This demonstrates that maintaining the original CFA sampling geometry improves not only pixel-wise fidelity and structural consistency, but also perceptual quality and color accuracy. The packed RGGB representation rearranges Bayer samples into four half-resolution channels, thereby altering the original spatial adjacency of CFA samples and weakening the correspondence between neighboring observations. In contrast, the proposed original-resolution representation preserves the CFA sampling geometry, enabling the network to more effectively exploit local structures and cross-position dependencies. This property is particularly important for region-level non-local modeling and residual aggregation, as both modules rely on accurate spatial relationships to aggregate structurally relevant features and refine high-frequency residual cues.

As shown in Table 8, the 2×2 region-level non-local strategy achieves the best overall reconstruction performance while requiring the shortest inference time. Compared with the global non-local operation, the region-level design not only improves reconstruction quality but also reduces inference-time computational cost. This demonstrates that modeling non-local dependencies within regional partitions can effectively alleviate the high computational burden of global non-local operations while preserving sufficient contextual information for image reconstruction. In contrast, using a finer partition does not further improve performance. It leads to inferior reconstruction quality and increased inference time, suggesting that excessive partitioning may weaken long-range dependency modeling and introduce additional overhead. Therefore, the 2×2 partition provides a better trade-off between reconstruction accuracy and computational efficiency and is adopted as the default setting in our method.

To further evaluate the effectiveness of the proposed residual aggregation strategy, we compare it with two commonly used feature fusion schemes, namely dense connections and skip connections, under the same experimental setting. As shown in Table 9, residual aggregation achieves the best performance across all evaluation metrics, demonstrating its superiority in both reconstruction accuracy and perceptual quality. Dense connections may introduce redundant feature responses by repeatedly concatenating multi-level features, while skip connections directly transmit shallow features without sufficient selection and refinement. In contrast, the proposed residual aggregation strategy explicitly collects and refines early-stage residual cues, which are closely related to missing color information, edge structures, and high-frequency details in demosaicking. By aggregating these residual compensation cues in a targeted manner, the network can more effectively recover fine textures and suppress reconstruction artifacts, thereby achieving more accurate and visually faithful demosaicking results.

### 3.5. Complexity Analysis

To evaluate the efficiency of the proposed RNRA-Net, we compare its model complexity and reconstruction performance with several representative demosaicking methods on the Kodak dataset. As shown in Table 10, RNRA-Net has the smallest number of parameters among all compared methods while achieving the best reconstruction performance. Compared with existing methods, RNRA-Net significantly reduces model complexity without sacrificing accuracy. These results indicate that the proposed method achieves a favorable balance between efficiency and performance, demonstrating its potential for practical deployment, especially in resource-constrained scenarios.

## 4. Discussion

The experimental results demonstrate that, on Kodak, McMaster, and City100 datasets, RNRA-Net achieves a superior overall balance among pixel-level fidelity, structural consistency, perceptual quality, and chromatic accuracy, with particularly pronounced advantages in edge-rich regions and repetitive texture areas. Compared with U-Net++, DPIR, and KLAP, RNRA-Net better satisfies the dual requirements of structural preservation and contextual modeling in demosaicking. In comparison with DTDeMo, the results further suggest that jointly modeling contextual dependencies and residual compensation within a unified end-to-end framework can be more effective than a two-stage local compensation pipeline. Therefore, the performance gain of RNRA-Net should not be regarded merely as an architectural improvement. It further indicates that respecting the structural constraints of the acquisition process is crucial for achieving high-quality demosaicking.

The present study is still mainly limited to the standard Bayer demosaicking setting, and its robustness under other CFA patterns and more realistic degradation conditions has not yet been fully validated. Future work will therefore focus on extending the proposed framework to other CFA layouts, multispectral demosaicking, and joint demosaicking-denoising tasks, while further exploring its applicability to broader image restoration problems. In addition, to meet the practical demands of real imaging pipelines, further lightweight redesign and hardware-oriented optimization will also be necessary.

## 5. Conclusions

This paper presents RNRA-Net, an end-to-end image demosaicking network that employs region-level non-local modeling and residual aggregation for high-quality color image reconstruction. The proposed network was designed to address the limitations of existing demosaicking methods in modeling cross-position correlations and effectively exploiting early-stage compensation cues. Specifically, the original mosaic image was represented as a three-channel input at the original resolution, thereby preserving the CFA spatial arrangement more faithfully than packed half-resolution representations. In addition, a region-level non-local module was introduced to establish feature correlations within an appropriate neighborhood, enabling more accurate extraction of contextual information beneficial to reconstruction. Furthermore, a residual aggregation strategy was developed to fuse and reinforce the relatively pure residual cues across different residual blocks, thereby improving feature-extraction efficiency and enhancing the recovery of edges, textures, and high-frequency details. Extensive experiments demonstrated that the proposed method outperformed existing approaches in both quantitative performance and visual quality. Ablation studies further verified the effectiveness of the proposed region-level non-local modeling and residual aggregation strategies. Future work will focus on extending the proposed framework to more challenging settings, including joint demosaicking and denoising, as well as lightweight deployment for practical imaging systems.

## Figures and Tables

**Table 1:** Comparison of underlying modeling mechanisms in demosaicking methods.

Method	Input Representation	Correlation Modeling	High-Frequency Feature Usage
DPIR	Degradation-model-based input	Plug-and-play denoiser prior	Generic restoration prior
U-Net++	Packed four-channel Bayer representation	Multi-scale convolutional fusion	Encoder–decoder detail fusion
KLAP	Unified CFA-pattern representation	Pattern-conditioned filtering	Filter-based color reconstruction
DTDeMo	Interpolation-based representation	Reconstruction refinement	Post-stage detail compensation
RNRA-Net	CFA-preserving full-resolution representation	Region-level non-local correlation	Explicit residual-compensation aggregation

**Table 2:** Summary of implementation setting of different methods.

Method	Input Representation	Implementation Setting/Source
U-Net++	Packed four-channel Bayer input	Method-specific settings follow released code
DPIR	Degradation-model-based input	Official plug-and-play demosaicking inference
KLAP	Unified CFA-pattern representation	Official pretrained model
DTDeMo	Interpolation-based representation	Method-specific settings follow the paper as closely as possible
RNRA-Net	Three-channel original-resolution CFA-preserving input	Settings described in Section 3.1

**Table 3:** Quantitative comparison on the Kodak and McMaster datasets (the bold indicates the best result, and the underlined denotes the suboptimal results).

Dataset	Model	PSNR	SSIM	LPIPS	CIEDE2000
Kodak	Bilinear	29.45	0.8901	0.1350	2.7953
	U-Net++	41.33	0.9873	0.0081	0.9109
	DPIR	40.61	0.9822	0.0147	1.0924
	KLAP	42.51	0.9902	0.0053	0.9919
	DTDeMo	43.14	0.9914	**0.0050**	1.0998
	RNRA-Net	**44.31**	**0.9934**	0.0051	**0.8022**
McMaster	Bilinear	31.33	0.9312	0.0742	2.5136
	U-Net++	37.71	0.9645	0.0068	1.8705
	DPIR	37.83	0.9663	0.0108	1.7386
	KLAP	39.81	0.9741	0.0057	1.5872
	DTDeMo	39.91	0.9664	0.0051	1.6310
	RNRA-Net	**40.02**	**0.9851**	**0.0048**	**1.4774**

**Table 4:** Quantitative comparison on the City100 dataset (the bold indicates the best result, and the underlined denotes the suboptimal results).

Dataset	Model	PSNR	SSIM	LPIPS	CIEDE2000
City100	Bilinear	31.19	0.9318	0.1161	2.4097
	U-Net++	36.78	0.9667	0.0079	1.5264
	DPIR	41.95	0.9859	0.0159	0.9438
	KLAP	42.72	0.9902	0.0071	0.8159
	DTDeMo	43.94	0.9911	0.0049	0.7899
	RNRA-Net	**44.60**	**0.9917**	**0.0047**	**0.7054**

**Table 5:** The ablation results of various modules on the Kodak dataset (the bold indicates the best result).

Model	Baseline	Residual Aggregation Module	Shared Skip Connection	Region-Level Non-Local Module	PSNR/SSIM/LPIPS
Model 1	✓	–	–	–	42.12/0.9892/0.0056
Model 2	✓	✓	–	–	42.37/0.9894/0.0052
Model 3	✓	–	–	✓	42.34/0.9892/0.0054
Model 4	✓	✓	✓	–	42.78/0.9899/**0.0050**
Model 5	✓	✓	–	✓	42.66/0.9895/0.0051
RNRA-Net	✓	✓	✓	✓	**44.31**/**0.9934**/0.0051

**Table 6:** The comparison of reconstruction performance with different numbers of blocks on the Kodak dataset (the bold indicates the best result).

Metric	1	2	3	4
PSNR	42.30	42.70	**44.31**	42.73
SSIM	0.9893	0.9898	**0.9934**	0.9899
LPIPS	0.0064	0.0059	**0.0051**	0.0054

**Table 7:** The comparison of reconstruction performance with different preprocessing on the Kodak dataset (the bold indicates the best result).

	PSNR	SSIM	LPIPS	CIEDE2000
Packed RGGB	37.18	0.9759	0.0218	1.5127
Ours	**44.31**	**0.9934**	**0.0051**	**0.8022**

**Table 8:** The comparison of reconstruction performance with different partitioning strategies on the Kodak dataset (the bold indicates the best result). Inference time denotes the average processing time per image.

	PSNR	SSIM	LPIPS	CIEDE2000	Inference Time (ms)
1 × 1 global	38.91	0.9819	0.0093	1.4660	17.046
2 × 2 (Ours)	**44.31**	**0.9934**	**0.0051**	**0.8022**	**10.457**
4 × 4	37.77	0.9828	0.0128	1.5453	14.525

**Table 9:** The comparison of reconstruction performance with different feature fusion schemes on the Kodak dataset (the bold indicates the best result).

	PSNR	SSIM	LPIPS	CIEDE2000
Dense connections	37.07	0.9765	0.0191	2.2459
Skip connections	38.21	0.9787	0.0161	1.5322
Residual aggregation (Ours)	**44.31**	**0.9934**	**0.0051**	**0.8022**

**Table 10:** Comparison of model complexity and performance on the Kodak dataset. Inference time denotes the average processing time per image.

Model	U-Net++	DPIR	KLAP	DTDeMo	RNRA-Net
Parameters (M)	2.80	17.90	7.28	1.26	0.88
FLOPs (G)	113.84	727.78	295.00	51.23	35.78
Inference time (ms)	33.27	212.71	86.51	14.97	10.46
PSNR	41.33	40.61	42.51	43.14	44.31

## Data Availability

The data will be made publicly available on reasonable request.
